# Diverging levels of COVID-19 governmental response satisfaction across middle eastern Arab countries: a multinational study

**DOI:** 10.1186/s12889-022-13292-9

**Published:** 2022-05-05

**Authors:** Rania Itani, Samar Karout, Hani M. J. Khojah, Makram Rabah, Mohamad B. Kassab, Francine K. Welty, Mazen AlBaghdadi, Haitham Khraishah, Faris El-Dahiyat, Salman Alzayani, Yousef S. Khader, Mohammad S. Alyahya, Danah Alsane, Rana Abu-Farha, Tareq L. Mukattash, Tarek Soukarieh, Mohamad Fawzi Awad, Reem Awad, Abir Wehbi, Fatima Abbas, Hadi El Mais, Huda El Mais, Lina Karout

**Affiliations:** 1 Pharmacy Practice Department, Faculty of Pharmacy, Beirut Arab University, Beirut, Lebanon; 2 Department of Clinical and Hospital Pharmacy, College of Pharmacy, Taibah University, Madinah, Kingdom of Saudi Arabia; 3 Department of History and Archeology, Faculty of Arts and Sciences, American University of Beirut, Beirut, Lebanon; 4 Division of Cardiology, Department of Medicine, Massachusetts General Hospital, Boston, USA; 5 Division of Cardiovascular Medicine, Department of Internal Medicine, Beth Israel Deaconess Medical Center, Harvard Medical School, Boston, MA USA; 6 Division of Cardiology, University of Maryland Medical Center, Baltimore, MD USA; 7 Clinical Pharmacy Program, College of Pharmacy, Al-Ain University, Al Ain, United Arab Emirates; 8 Department of Family and Community Medicine, College of Medicine and Medical Sciences, Arabian Gulf University, Manama, Kingdom of Bahrain; 9 Epidemiology, Medical Education and Biostatistics, Department of Community Medicine, Public Health and Family Medicine, Faculty of Medicine, Jordan University of Science & Technology, Irbid, Jordan; 10 Department of Health Management and Policy, Faculty of Medicine, Jordan University of Science and Technology, Irbid, Jordan; 11 Department of Pharmacy Practice, Faculty of Pharmacy, Kuwait University, Kuwait, Kuwait; 12 Clinical Pharmacy and Therapeutics Department, Faculty of Pharmacy, Applied Science Private University, Amman, Jordan; 13 Department of Clinical Pharmacy, Faculty of Pharmacy, Jordan University of Science and Technology, Irbid, Jordan; 14 American University of Beirut Medical Center, Beirut, Lebanon; 15 Health Department, United Nations Relief and Works Agency for Palestine Refugees in the Near East (UNRWA), Beirut, Lebanon; 16 Department of Immunology, Faculty of Arts and Science, University of Toronto, Toronto, Ontario Canada; 17 Faculty of Medicine, Beirut Arab University, Beirut, Lebanon

**Keywords:** Government, Perception, Middle East, Arab countries, COVID-19, Level of satisfaction

## Abstract

**Background:**

Public acceptance of governmental measures are key to controlling the spread of infectious diseases. The COVID-19 pandemic has placed a significant burden on healthcare systems for high-income countries as well as low- and middle-income countries (LMICs). The ability of LMICs to respond to the challenge of the COVID-19 pandemic has been limited and may have affected the impact of governmental strategies to control the spread of COVID-19. This study aimed to evaluate and compare public opinion on the governmental COVID-19 response of high and LMICs in the Middle East and benchmark it to international countries.

**Methods:**

An online, self-administered questionnaire was distributed among different Middle Eastern Arab countries. Participants’ demographics and level of satisfaction with governmental responses to COVID-19 were analyzed and reported. Scores were benchmarked against 19 international values.

**Results:**

A total of 7395 responses were included. Bahrain scored highest for satisfaction with the governmental response with 38.29 ± 2.93 on a scale of 40, followed by the Kingdom of Saudi Arabia (37.13 ± 3.27), United Arab Emirates (36.56 ± 3.44), Kuwait (35.74 ± 4.85), Jordan (23.08 ± 6.41), and Lebanon (15.39 ± 5.28). Participants’ country of residence was a significant predictor of the satisfaction score (*P <* 0.001), and participants who suffered income reduction due to the pandemic, had a history of SARS-CoV-2 infection, and held higher educational degrees had significantly lower satisfaction scores (*P <* 0.001). When benchmarked with other international publics, countries from the Gulf Cooperation Council had the highest satisfaction level, Jordan had an average score, and Lebanon had one of the lowest satisfaction scores.

**Conclusion:**

The political crisis in Lebanon merged with the existing corruption were associated with the lowest public satisfaction score whereas the economical instability of Jordan placed the country just before the lowest position. On the other hand, the solid economy plus good planning and public trust in the government placed the other countries of the Gulf Cooperation Council on top of the scale. Further investigation is necessary to find out how the governments of other low-income countries may have handled the situation wisely and gained the trust of their publics. This may help convey a clearer picture to Arab governments that have suffered during the pandemic.

## Introduction

The World Health Organization declared a global health emergency on March 11, 2020, due to the new coronavirus (SARS-CoV-2) responsible for the COVID-19 disease [1–3]. In the wake of the global health emergency resulting from the COVID-19 pandemic, enormous pressure was placed on healthcare systems worldwide, exposing internal, structural, and functional gaps in different organizations deployed by governments [4]. The global lack of preparedness for such a pandemic was prominent initially, generating fear and dread in the society [5]. The pandemic revealed multiple fault lines in communities, economies, and healthcare institutions worldwide which prompts many Arab nations to implemented pandemic response plans to overcome these challenges by formulating health policies, laws, and strategies to limit COVID-19 spread [6]. As of September 3, 2021, the Middle East had reported over 19,228,148 COVID-19 cases, including a total of 2,483,113 cases from the Gulf Cooperation Council countries, while Lebanon and Jordan together reported 1,401,379 cases [7].

Public adherence to preventive measures and obedience to government instructions significantly impacted the course of the pandemic, incidence, and fatality rate among different nations [8, 9]. Possible reasons for nonadherence may include the lack of trust in governments, the implementation of ineffective strategies to contain the pandemic, and the lack of effective national communication about COVID-19 [10]. Governmental trust and community compliance to preventive measures are strongly correlated, as revealed during the H1N1 pandemic and the Ebola outbreak [10, 11]. Thus, an emerging pandemic cannot be controlled without broad public support. Furthermore, evidence-based communication promotes transparency and confidence, enabling the population and government officials to make informed decisions [12, 13].

Understanding the publics’ perceptions and the factors behind their non-compliance would aid in fostering public cooperation. Therefore, the current study aimed to investigate and compare the public satisfaction with governmental responses in handling the COVID-19 pandemic in different Middle Eastern Arab countries. We also aimed to identify the critical predictors associated with their satisfaction towards the governmental responses, potentially delivering a comprehensive view of disease control strategic plans for future pandemics. Moreover, our results will be benchmarked against other international findings.

## Materials and methods

### Study design

This observational cross-sectional study was conducted using an online self-administered questionnaire between April 1, 2021, and April 30, 2021. The link to the survey form was distributed through different social networking platforms (WhatsApp, Twitter, Facebook, and Instagram), as well as multiple news and radio platforms helped in sharing and inviting participants to fill the survey. This survey tageted adults (≥ 18 years) residing in different Middle Eastern Arab countries. The survey was available in English and Arabic, according to the preference of the participants.

### Questionnaire development and structure

The research team developed the questionnaire after an extensive literature review of relevant studies [8, 12]. The questionnaire consisted of 38 items, varying between closed-ended questions (with pre-defined answers) and open-ended ones. This questionnaire was mainly divided into two main sections. The first section was dedicated to retrieving participants’ socio-demographic data, while the second recorded their satisfaction with their governmental responses in handling the COVID-19 pandemic. The latter section consisted of a well-validated and reliable tool, adapted from Lazarus et al., upon their approval [8]. This tool comprised eight items, each measuring a different area of participant satisfaction with the governmental response during the pandemic on a 5-point Likert scale (1 = Strongly disagree, 2 = disagree, 3 = neutral, 4 = agree, and 5 = strongly agree). Points from each item were added up to get a score out of 40. The total score values were multiplied by 100 to allow for comparison with Lazarus et al.’s results to benchmark the COVID-19 score in Middle Eastern countries with international ones [8]. Cronbach’s alpha test was used to test the internal reliability, which yielded 0.82, indicating that the scale was reliable with good internal consistency.

The questionnaire section covering the satisfaction with governmental response consisted of the following eight items:

1. Do you approve the way your country of residence is handling the pandemic?
2. Your country of residence helped you and your family meet your daily needs during the COVID-19 pandemic in terms of income and food.
3. Your country of residence communicated clearly to ensure that everyone had the information they needed to protect themselves and others from COVID-19, regardless of socioeconomic level, migrant status, ethnicity, or language.
4. You trust your country of residence’s reports on the spread of the pandemic and the statistics on the number of COVID-19 cases and deaths.
5. Your country of residence provided everyone with access to free, reliable COVID-19 testing if they had symptoms.
6. Your country of residence made sure you always had full access to the healthcare services you needed during the pandemic.
7. Your country of residence provided special protections to vulnerable groups at higher risk such as the elderly, the poor, migrants, prisoners, and the homeless during the COVID-19 pandemic.
8. Your country of residence provided mental health services to help people suffering from loneliness, depression, and anxiety caused by the COVID-19 pandemic.

### Questionnaire revision and piloting

The questionnaire was reviewed by medical and social research experts for the assessment of its content validity, relevance, specificity, and comprehensiveness. Then, pilot testing was employed on a convenience sample of 40 participants, where they were requested to fill out the self-administered questionnaire, and provide feedback on its understandability, clarity, cultural acceptability, and length. As such, some questions from the original COVID-SCORE-10 [8] were remodeled based on respondents’ feedback, and three additional questions were added to tackle the purpose of the study. These questions were: “Do you trust the government to successfully address unexpected health threats related to the COVID-19 pandemic?”, “Where does your country stand during the pandemic?”, and “Do you feel that the government neglected the residing foreigners and undocumented immigrants during this pandemic?”. 1 week later, the questionnaire was retested on the same participants to ensure its reliability and reproducibility. Data obtained from the pilot test was not included in the final data analysis.

### Ethical considerations

The study design and conduction followed the World Medical Association’s Declaration of Helsinki guidance. The study was approved by the Research and Ethics Committee at Beirut Arab University (No. 2020-H-0071-P-R-0435). The aim of the study was explained in the introduction of the questionnaire and the participants were requested to approve an electronic informed consent, which contained a statement about anonymity of the survey, voluntary participation, and the right to defer from submitting their responses at any time. The anonymity of respondents was preserved, as the participants’ names, personal data, or any identifiers were not collected.

### Statistical analysis

Data were analyzed by SPSS (New York, USA). The mean ± standard deviation (SD), and frequency (or percentages) were used for continuous and categorical variables, respectively. Univariate and multiple linear regression were used to screen for predictors of the satisfaction with governmental response scores. All variables with *p <* 0.25 resulting from the univariate linear analysis were entered into a multiple linear regression model, using backward stepwise analysis. Results with a *p* ≤ 0.05, with a 95% confidence interval, were considered significant.

## Results

### Socio-demographic characteristics

A total of 7395 individuals responded to the survey, including 2385 (36%) from Lebanon, 1965 (29.7%) from Kuwait, 1609 (24.3%) from Bahrain, 773 (10.5%) from Jordan, 388 (5.9%) from the Kingdom of Saudi Arabia (KSA), and 275 (4.2%) from the United Arab Emirates (UAE). The socio-demographic data of the participants from the six countries are summarized in Table 1. There were significant differences in all socio-demographic variables between the six countries (*p*-value < 0.001). The mean age of the study participants was 39.82 ± 15, while almost half of the participants were adults younger than 35 years (3183, 43.0%), and almost 60% of study participants were married (4290, 58%). The majority of respondents were women (68.7%). Of the respondents, 94% were citizens of the country they were living in at the time of survey administration. More than two-thirds of all respondents had a university degree, and 18 of the 26 illiterate respondents were from Lebanon (69.2%). Lebanon also had the lowest household income, in which 70.4% (*n =* 870) of the participants who reported having a monthly household income of less than $500 were Lebanese. Respondents reporting a monthly household income of more than $5000 were predominantly from Kuwait 864 (44%). Among the 3752 employed participants, 57.4% (*n =* 2152) reported changes in their current jobs attributed to either the COVID-19 pandemic, the economic crisis, or both. Furthermore, salary reduction was one of the main consequences experienced by 32.1% (*n =* 3752) of the employed participants.

**Table 1:** Socio-demographic data of the study participants from the six countries (*N =* 7395)

Characteristic	Total (***N =*** 7395)^**a**^	Lebanon (***n =*** 2385)^**b**^	Kuwait (***n =*** 1965)^**b**^	Bahrain (***n =*** 1609)^**b**^	Jordan (***n =*** 773)^**b**^	KSA (***n =*** 388)^**b**^	UAE (***n =*** 275)^**b**^	P^**c**^
**Age** (years, Mean ± SD)	39.82 ± 15	28.92 ± 10	50.13 ± 12	47.02 ± 12	33.29 ± 11	41.3 ± 13	34.7 ± 13	< 0.001
< 35	3183 (43.0)	1867 (78.3)	287 (14.6)	313 (19.5)	442 (57.2)	121 (31.2)	153 (55.6)	< 0.001
35–49	1925 (26)	365 (15.3)	546 (27.8)	536 (33.3)	258 (33.4)	146 (37.6)	74 (26.9)	
50–64	1915 (25.9)	136 (5.7)	908 (46.2)	655 (40.7)	64 (8.3)	112 (28.9)	40 (14.5)	
≥ 65	372 (5)	17 (0.7)	224 (11.4)	105 (6.5)	9 (1.2)	9 (2.3)	8 (2.9)	
**Sex** (%)								< 0.001
Men	2316 (31.3)	688 (28.8)	516 (26.3)	538 (33.4)	245 (31.7)	217 (55.9)	112 (40.7)	
Women	5079 (68.7)	1697 (71.2)	1449 (73.7)	1071 (66.6)	528 (68.3)	171 (44.1)	163 (59.3)	
**Citizenship** (%)								
Citizen	6950 (94)	2323 (97.2)	1915 (97.4)	1557 (96.8)	754 (97.5)	283 (72.9)	118 (42.9)	
Resident	445 (6)	62 (2.6)	50 (2.5)	52 (3.2)	19 (2.5)	105 (27.1)	157 (57.1)	< 0.001
**Education Level** (%)								
No education/Illiterate	26 (4)	18 (0.8)	1 (0.1)	2 (0.1)	5 (0.6)	0 (0)	0 (0)	< 0.001
School Degree	848 (11.5)	353 (14.8)	133 (6.8)	203 (12.6)	101 (13.1)	43 (11.1)	15 (5.5)	
Some College	1132 (15.3)	243 (10.2)	400 (20.4)	306 (19)	72 (9.3)	48 (12.4)	63 (22.9)	
Bachelor’s Degree	3703 (50.1)	1000 (41.9)	1201 (61.1)	694 (43.1)	462 (59.8)	183 (47.2)	163 (59.3)	
Post Graduate Degree	1686 (22.8)	771 (32.3)	230 (11.7)	404 (25.1)	133 (17.2)	114 (29.4)	34 (12.4)	
**Marital Status** (%)								
Single/Engaged	2656 (35.9)	1623 (68.1)	280 (14.2)	219 (13.6)	318 (41.1)	84 (21.6)	132 (48)	< 0.001
Married	4290 (58)	697 (29.2)	1470 (74.8)	1265 (78.6)	431 (55.8)	294 (75.8)	133 (48.3)	
Widowed/Divorced	449 (6.1)	65 (2.7)	215 (10.9)	125 (7.8)	24 (3.1)	10 (2.6)	10 (3.6)	
**Monthly household income** (%)								
< $500	1236 (16.7)	870 (54.7)	12 (0.6)	67 (4.2)	258 (40.4)	17 (4.4)	12 (4.4)	< 0.001
$501 - $1000	865 (11.7)	292 (18.4)	97 (4.9)	201 (12.5)	233 (36.5)	23 (5.9)	19 (6.9)	
$1001 - $2000	807 (10.9)	167 (10.5)	192 (9.8)	287 (17.8)	105 (16.4)	36 (9.3)	20 (7.3)	
$2001 - $3000	720 (9.7)	85 (5.3)	256 (13)	254 (15.8)	24 (3.8)	60 (15.5)	41 (14.9)	
$3001 - $4000	612 (8.3)	43 (2.7)	267 (13.6)	203 (12.6)	11 (1.7)	55 (14.2)	33 (12)	
$4001 - $5000	562 (7.6)	3 (0.ƒ2)	277 (14.1)	185 (11.5)	2 (0.3)	62 (16)	33 (12)	
> $5000	1664 (22.5)	130 (8.2)	864 (44)	412 (25.6)	6 (0.9)	135 (83.8)	117 (42.5)	
**Employment Status** (%)								
No	3643 (49.3)	1097 (46)	1031 (52.5)	835 (51.9)	392 (50.7)	138 (35.6)	150 (54.5)	< 0.001
Yes	3752 (50.7)	1288 (54)	934 (47.5)	774 (48.1)	381 (49.3)	250 (64.4)	125 (45.5)	

*KSA* Kingdom of Saudi Arabia, *UAE* United Arab Emirates.

^a^ Percentages for columns

^b^ Percentages for rows

^c^ Significance at *p ≤* 0.05

Upon questioning the participants with the following “Where does your country of residents stand in this COVID-19 pandemic?”, more than half of them (58.7%, *n =* 4344) believed that they had passed the most difficult time, while 41.3% (*n =* 3051) assumed that the worst is yet to come. Moreover, 35.7% (*n =* 2639) of the participants reported that the government neglected foreigners and undocumented immigrants residing in their countries during this pandemic.

### Satisfaction score of governmental response

Table 2 summarizes the scores of the satisfaction with government responses against COVID-19 as reported by the participants from the six Arab countries. The Gulf Cooperation Council countries had the highest scores, with Bahrain in the lead, followed by KSA, UAE, and Kuwait. On the other hand, Jordan and Lebanon had the lowest satisfaction with governmental response scores of 23.08 ± 6.41 and 15.39 ± 5.28, respectively. The highest-scoring item (3.92 ± 1.36) across all countries was related to the governmental communication with the public about the pandemic and the preventive measures, while the lowest-scoring item was related to the mental health services provided. Lebanon scored the lowest grade among the Arab countries related to the provision of basic daily needs (1.48 ± 0.79), and Bahrain scored the highest grade related to access to free COVID-19 testing (4.93 ± 0.32).

**Table 2:** Scores^a^ of governmental responses against COVID-19

Score items	Total ***N =*** 7395 (100%)	Lebanon***n =*** 2385 (32.3%)	Kuwait***n =*** 1965 (26.6%)	Bahrain ***n =*** 1609 (21.8%)	Jordan ***n =*** 773 (10.5%)	KSA***n =*** 388 (5.2%)	UAE ***n =*** 275 (3.7%)	***P***^**b**^
Do you approve the way your country of residence is handling the pandemic?	3.51 ± 1.53	1.88 ± 0.95	4.4 ± 0.85	4.81 ± 0.52	2.54 ± 1.09	4.78 ± 0.52	4.59 ± 0.61	< 0.001
Your country of residence helped you and your family meet your daily needs during the COVID-19 pandemic in terms of income and food	3.51 ± 1.71	1.48 ± 0.79	4.82 ± 0.47	4.88 ± 0.44	2.48 ± 1.16	4.80 ± 0.42	4.72 ± 0.50	< 0.001
Your country of residence communicated clearly to ensure that everyone had the information they needed to protect themselves and others from COVID-19, regardless of socioeconomic level, migrant status, ethnicity, or language	3.92 ± 1.36	2.56 ± 1.22	4.73 ± 0.62	4.9 ± 0.39	3.28 ± 1.13	4.84 ± 0.43	4.73 ± 0.49	< 0.001
You trust your country of residence’s reports on the spread of the pandemic and the statistics on the number of COVID-19 cases and deaths	3.62 ± 1.42	2.39 ± 1.15	4.31 ± 0.97	4.76 ± 0.57	2.66 ± 1.16	4.36 ± 0.91	4.42 ± 0.82	< 0.001
Your country of residence provided everyone with access to free, reliable COVID-19 testing if they had symptoms	3.67 ± 1.54	1.86 ± 1.02	4.55 ± 0.78	4.93 ± 0.32	3.51 ± 1.09	4.82 ± 0.47	4.67 ± 0.54	< 0.001
Your country of residence made sure you always had full access to the healthcare services you needed during the pandemic	3.67 ± 1.49	1.98 ± 1.01	4.58 ± 0.73	4.89 ± 0.37	3.23 ± 1.07	4.73 ± 0.53	4.63 ± 0.63	< 0.001
Your country of residence provided special protections to vulnerable groups at higher risk such as the elderly, the poor, migrants, prisoners, and the homeless during the COVID-19 pandemic	3.51 ± 1.57	1.67 ± 0.82	4.53 ± 0.76	4.80 ± 0.50	2.93 ± 1.082	4.63 ± 0.62	4.63 ± 0.59	< 0.001
Your country of residence provided mental health services to help people suffering from loneliness, depression, and anxiety caused by the COVID-19 pandemic	3.10 ± 1.48	1.59 ± 0.80	3.83 ± 1.07	4.31 ± 0.88	2.45 ± 1.03	4.18 ± 0.87	4.18 ± 0.80	< 0.001
**Total Score (out of 40, mean ± SD)**	28.51 ± 11.04	15.39 ± 5.28	35.74 ± 4.85	38.29 ± 2.93	23.08 ± 6.41	37.13 ± 3.27	36.56 ± 3.44	< 0.001
**Total Score (out of 100, mean ± SD)**	71.28 ± 27.60	38.48 ± 13.20	89.35 ± 12.13	95.73 ± 7.33	57.70 ± 16.03	92.83 ± 8.18	91.40 ± 8.60	< 0.001

*KSA* Kingdom of Saudi Arabia, *SD* Standard deviation, *UAE* United Arab Emirates.

^a^ 5-point Likert Scale coded as: strongly disagree = 1, disagree = 2, neutral = 3, agree = 4, and strongly agree = 5

^b^ Significance at *p ≤* 0.05

### Predictors of the satisfaction score of governmental response

Table 3 reveals the significant predictors influencing the satisfaction with governmental response score, using linear regression analysis. Participants older than 50 years reported a statistically significant higher satisfaction with governmental response scores (*p <* 0.001). Similarly, residents reported a significantly higher score compared with citizens. In contrast, experiencing salary reduction, being previously infected with COVID-19, and having a higher educational degree, were significantly associated with a lower score.

**Table 3:** Predictors of the satisfaction with governmental response score

Model	Simple linear regression		Multiple linear regression	
	Beta	***P***^**a**^	Beta	***P***^**a**^
**Citizenship**				
Citizen	Reference			
Resident	0.088	< 0.001^b^	0.107	< 0.001
**Country**	−1.17	0.05	−0.37	< 0.001
**Sex**				
Men	Reference			
Women	0.574	0.566	–	–
**Age (years)**	0.539	< 0.001^b^	0.537	< 0.001
**Education**				
Diploma or school level	Reference			
University or graduate degrees	−0.039	< 0.001^b^	−0.053	< 0.001
**Income decreased**				
No	Reference			
Yes	−0.137	< 0.001^b^	−0.102	< 0.001
**Previously infected with COVID-19**				
No	Reference			
Yes	−0.179	< 0.001^b^	− 0.172	< 0.001

^a^ Significance at *p ≤* 0.05. All variables with *p <* 0.25 resulting from the univariate linear analysis were entered into a multiple linear regression model using backward stepwise analysis

^b^ Eligible for entry in multiple linear regression

### Benchmarking public satisfaction scores with international publics

**Fig. 1:**
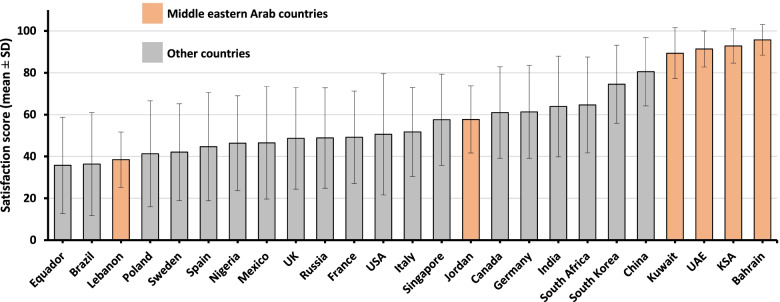
Benchmarking of Middle Eastern versus international public satisfaction with governmental responses against COVID-19. *KSA*, Kingdom of Saudi Arabia; *SD*, standard deviation; *UAE*, United Arab Emirates; *UK*, United Kingdom; *USA*, United States of America

In Fig. 1, the six countries of our study were plotted along with the nineteen countries Lazarus et al. studied. The figure shows that the Gulf Cooperation Council countries were the highest scoring out of all 25 countries included. Jordan’s scores stood near the middle amongst most countries, and Lebanon’s were third from the lowest values.

## Discussion

Public approval is a key element of a successful pandemic response [8, 13]. COVID-19 has exposed Governments around the world to a range of unique challenges that can and cannot be foreseen but that are best mitigated by a coordinated and communicated approach [8, 13]. The pandemic and the associated government responses have emerged as potential measuring sticks for the acceptance of statehood and good governance. Countries with greater stability and resources to address immediate testing and critical care needs could theoretically keep their citizens more satisfied and ultimately responsive to the public health measures and lockdowns needed to control the pandemic [14]. Therefore, public opinion represents a clear reflection of the governmental responses to the pandemic. This study aimed to assess and compare the level of satisfaction on the COVID-19 governmental response of six Middle Eastern countries with different socio-economical statuses. Interestingly, our results showed that residents of countries experiencing economic crisis and conflicts are dissatisfied with the COVID-19 response of their countries whereas, residents of countries with better economy are very satisfied with the COVID-19 governmental response.

Among the six countries surveyed in this study, Lebanon was the lowest on the governmental satisfaction scale as opposed to the highest score that went to Bahrain, followed by KSA, UAE, Kuwait, and Jordan. The significant variability in the satisfaction scores across the six countries was anticipated given the prominent differences in socio-economic statuses, political stability, as well as the implemented vaccination nationalism.

The Levant countries, in the Eastern Mediterranean region, which includes Lebanon, Syria, Palestine, Iraq, and Jordan are countries with conflict zone and politically fragmented areas, putting the governments at a disadvantageous position in responding to the new portfolio in controlling the emerging pandemic [15]. The pandemic has further aggravated the situation and created an extreme burden on countries with economic despair and vulnerable healthcare system [16]. In addition, due to the absence of a robust and efficient healthcare system in the low to middle-income countries, the presence of a high rate of uninsured citizens, the lack of national health authorities’ supervision, the healthcare system became saturated, with the inability of hospitals to serve all patients who required medical services [15, 17–22]. On the other hand, health care providers faced tremendous challenges in terms of securing medical supplies and receiving financial compensation to carry out a role beyond their traditional role [3, 9, 22–29]. Many of them were constantly harassed, physically and mentally abused [30, 31].

It is prejudicial to compare Lebanon to wealthier neighboring such as the Gulf Cooperation Council countries. Lebanon’s low approval rating score can be directly linked to its political and economic crises, which predated the outbreak of the pandemic [32]. Lebanon has been assailed by multifaceted crises, economic crises, political instability, regional conflict, extreme poverty, and lastly the port explosion on August 4, 2020 [33, 34]. It is worth mentioning that Lebanon also hosts the largest number of Syrian refugees per capita, placing enormous pressure on the country’s already deteriorated economy and financial climate [35]. Since 2019, around 80% of the Lebanese population has fallen into poverty. The World Bank declared that it is one of the worst miseries of modern times. The Lebanese currency has fallen more than 90% of its value, defeating purchasing ability in a nation dependent on imports. Furthermore, the Lebanese banking system has collapsed, with depositors unable to cash withdraw their foreign currency savings and forced to draw out cash in the collapsing local currency (the Lebanese Lira) [36, 37]. The total financial and political collapse was made even worse with the spread of SARS-CoV-2 infection, which reached Lebanon by the end of February 2020 [38]. Additionally, the significant degree of corruption across the country was a solid foundation for distrust between the people and government. The government’s response to the pandemic lacked basic components needed for control of the spiking COVID-19 cases. Contributing factors include, but are not limited to, inadequate lockdowns, failure to place strict regulations, and delayed vaccine availability.

Today, Lebanon continues to face one of the worst political and economic crises worldwide, with basic food and medicine shortages and a daily fluctuation of the Lebanese currency [23, 39–42]. The government’s lack of proper measures was directly related to the escalation of COVID-19 cases in the country; the 7-day average of new cases almost doubled in the 40 days of our survey administration [24]. Furthermore, at the time of our survey, Lebanon had experienced its highest cases and deaths and reached over 520,000 cases (7.6% cases for the population ~ 6.8 million) and 7300 deaths (1.4%) [24].

Similarly, Jordan was also experiencing some economic instability before the spread of the pandemic [43]. Left with a few choices, Jordan adopted strict measures such as an extended curfew, heavy penalties to violators, and business closures, comparable to the procedures implemented in more economically stable countries, e.g., the Gulf Cooperation Council countries. This allowed Jordan to buy time until the vaccine became nationally available. It is worth mentioning that vaccine nationalization was of a global concern where high-income countries have raced to invest in the stock of vaccinations, which in turn has led to inequity of vaccine supply and distribution in low and middle-income countries [26, 44, 45]. However, despite the innovative science-based approach employed by the new Jordanian government, the economic challenges were hard to face [46]. Concurrently, the medical field faced additional difficulties, and several COVID-19 patients died due to oxygen shortage, which kept the country at unease [47]. Furthermore, at the time of our survey, Jordan had over 700,000 confirmed cases (6.8% of a population ~ 10.3 million) and 8800 deaths (1.3%) [24].

On the other hand, the situation in the four Gulf Cooperation Council countries that scored highest (i.e., Bahrain, KSA, UAE, and Kuwait) was similar through the wise use of their resources and stable economies to alleviate the health and economic impact of COVID-19. Since the beginning of the outbreak, these countries adopted strict measures, including mandatory lockdowns in KSA, UAE, and Kuwait and non-mandatory lockdown in Bahrain. Traveling from and to these countries, except Bahrain, was prevented. In addition, these countries prohibited mass gathering and spread the concept of hygiene and social distancing through local and social media. Specific smartphone applications were employed to communicate between the health authorities and the public, and a work-from-home system was adopted [48, 49]. Moreover, treatment, quarantine, screening services, and vaccines were offered freely even for the residence violators, along with stabilizing food and medical commodities’ levels and market prices [16, 50–54]. In addition, at the time of our survey, the number of COVID-19 cases and deaths in UAE, KSA, Bahrain and Kuwait was over 520,000 cases (5.2% of population ~ 10 million) with 1590 deaths (0.3%), 418,000 cases (1.2% of population ~ 35.4 million) with 7000 deaths (1.7%), 178,000 cases (9.9% of population ~ 1.8 million) with 648 deaths (0.4%), and 275,000 cases (6.5% of population ~ 4.2 million) with 1570 deaths (0.6%), respectively [24].

In the current study, no clear relationship between the number of confirmed cases and the level of satisfaction in each country was noticed. Having lower confirmed COVID-19 cases did not reflect the actual prevalence as some countries, such as Lebanon, were not sufficiently testing for SARS-CoV-2 like the Gulf Cooperation Council countries. Interestingly, when conducting our study, the COVID-19 mortality rate was consistent with the governmental satisfaction score in all countries except in KSA. Lebanon had the second-highest mortality rate of 1.4%, with the lowest COVID-19 score (15.39 ± 5.28), followed by Jordan at 1.3% mortality rate and 23.08 ± 6.41 score, then Kuwait, which had a 0.6% mortality rate and a 35.74 ± 4.85 score followed by Bahrain and UAE which had the lowest mortality rates at 0.3–0.4% and scores among the highest (38.29 ± 2.93 and 36.56 ± 3.44, respectively). It can be deduced from our study findings that a lower governmental response score was associated with countries with higher mortality rates. This inference is in line with a previous study that concluded that government responses do indeed have a significant relationship with deaths related to COVID-19 [55]. On the other hand, KSA was an exception to this, where it had the highest mortality rate compared with all surveyed countries (1.7%) and the second-highest COVID-19 score (37.13 ± 3.27). This finding may be due to the low reported public adherence to safety measures in the country [16], despite the strict regulations that KSA put in place after undergoing a peak of cases early in the year 2020, which would explain the high levels of satisfaction regardless of how critical the COVID-19 situation was [56].

Although the scale of the economy of the studied countries may have influenced the six countries’ mean scores, ranging from extreme satisfaction to extreme dissatisfaction (i.e., Lebanon was well below the average at 15.39 ± 5.28 versus Bahrain, nearly at 100% satisfaction with a score of 38.29 ± 2.93)”, other factors may also have a significant impact. The key elements of public satisfaction appear to be related to a solid and stable governance infrastructure, the wise use and allocation of resources, public awareness, preparedness, and trust in the government [57].

In a previous study by Lazarus et al., the COVID-SCORE was developed, distributed, and validated across 19 countries worldwide [8]. We found consistent correlations between the factors and scores when comparing our results with that study. For example, a history of previous COVID-19 infection negatively correlated with satisfaction scores in both studies. Moreover, strict lockdown regulations and early vaccine demonstration in certain parts of Asia reflected higher satisfaction scores than some Latin, North American, and European countries. We observed similar results in our study with the Gulf Cooperation Council countries versus Lebanon and Jordan. Overall, developing countries included in the Lazarus et al. study showed lower population satisfaction scores which were analogous to our findings in disadvantaged countries in the Middle East region. Furthermore, consistent with what Lazarus et al. had described, the higher scores reported by people residing in the Gulf Cooperation Council countries also reflected higher levels of general trust in their public health experts than in Lebanon and Jordan.

Another interesting finding in our data was that residents had significantly higher mean scores than citizens of the same country. One potential factor is that most residents had fled their countries of origin, which are generally developing ones (such as Syria, Lebanon, India, and others) that are going through worse conditions than the countries of current residence. This factor would positively influence their perception of the current government’s actions amidst the pandemic compared to how its citizens perceive the situation.

Finally, although this study was proactive in investigating the Arab population’s perspectives toward their governments handling the COVID-19 pandemic, some limitations must be pointed out. First, this study included only six Arab countries, where findings may not be generalizable to the Arab world due to diversity in their economic status and political directions, although they share a prevailing culture. Second, we recruited a convenience sample of participants via social media, which may have introduced selection bias, limiting the generalizability of results to the general population. Third, the elderly population aged 65 years and above were underrepresented in our study sample, this could be interpreted to the nature of the web-based questionnaire. Fourth, the pandemic was not impacting all countries in the same way during the period of data collection, where UAE, Lebanon, Kuwait and KSA were under the third wave of the pandemic, Jordan was at the end of the second wave and Bahrain was just before the fourth wave. These differences could potentially result in different population perspectives on the governmental response. Fifth, some of the countries included in the study may have more restrictive media access which consequently would convey a message that is more favorable to the government. Another possible limitation is the low participation of men and non-citizens in the Gulf Cooperation Council countries, where many of these residents are labor workers who do not speak either Arabic or English.

## Conclusion

The COVID-19 pandemic has placed world governments under tremendous economic, logistical, and political pressures. It seems that the economic status plus the wise use of resources of each country played a major role in the COVID-19 response satisfaction, where high-income countries such as the Gulf Cooperation Council countries had the highest satisfaction rates worldwide. The satisfaction score of Jordan was significantly lower than the Gulf Cooperation Council countries, and Lebanon scored extremely low. Further studies may be required to investigate the factors that contributed to the high rate of satisfaction in the Gulf countries compared to other developed countries. On the other hand, the situation in other low-income countries that survived the pandemic with the least possible economic burden, and maintained the trust of their people, must be investigated. Hence, a clear message can be conveyed to the governments of those Arab countries that failed or suffered dealing with the pandemic, and eventually scored low for the public satisfaction.

## Data Availability

The dataset presented in this article is available only upon reasonable request since it contains confidential information. Requests to access the datasets should be directed to the corresponding authors.
